# Evaluating a Fusion Teaching-Learning Method for Enhancing Comprehension, Retention, and Clinical Application of ENT Surgical Anatomy in MBBS Students

**DOI:** 10.7759/cureus.88220

**Published:** 2025-07-18

**Authors:** Baddam Rachna Reddy, Gudise Abhinav Kiran, Shruthi Vadla, Yakaiah Vangoori

**Affiliations:** 1 Department of Otolaryngology, Malla Reddy Medical College for Women, Hyderabad, Hyderabad, IND; 2 Department of Otolaryngology, Malla Reddy Institute of Medical Sciences, Hyderabad, IND; 3 Department of Pharmacology, Malla Reddy Medical College for Women, Hyderabad, Hyderabad, IND

**Keywords:** clinical application and retention, competency-based medical education, constructivist medical pedagogy, ent surgical anatomy education, fusion teaching-learning method, multimodal instructional strategy, undergraduate medical education innovation

## Abstract

Background: Traditional methods of anatomy instruction often fall short in promoting clinically relevant, long-term understanding, particularly in surgical domains such as otorhinolaryngology (ENT), which require high-level spatial reasoning and procedural competence. This study aimed to evaluate the effectiveness of an innovative, multimodal Fusion Teaching-Learning Method (TLM), conceptualised as “From Pixels to Scalpels,” that integrates digital visualisation, hands-on engagement, and collaborative case-based tasks in enhancing the comprehension, retention, and clinical application of ENT surgical anatomy among MBBS students.

Methods: A prospective, crossover observational study was conducted involving 60 Phase III Part I MBBS students at a tertiary teaching institution. Participants experienced both traditional and Fusion TLM interventions across two ENT topics, with outcomes assessed through pre-, post-, and two-week retention multiple-choice question (MCQ) tests, rubric-based team assessments, and structured perception surveys. Statistical analysis included paired and unpaired t-tests for knowledge comparisons and descriptive analysis for student feedback.

Results: The Fusion TLM significantly improved post-test scores (p = 0.007 and 0.004), rubric-based application scores (p = 0.003 and 0.002), and knowledge retention (p = 0.003 and 0.001) compared to traditional methods. Over 90% of students reported enhanced engagement, conceptual clarity, and confidence, with 75% affirming improved self-efficacy and clinical reasoning abilities.

Conclusion: The “From Pixels to Scalpels” model demonstrated clear pedagogical superiority, combining cognitive, psychomotor, and affective domains to foster deeper learning and clinical relevance. Its scalability, learner-centred design, and alignment with Competency-Based Medical Education (CBME) make it a promising strategy for anatomical education in resource-constrained settings.

## Introduction

The ever-evolving demands of healthcare delivery call for a radical transformation in the way future clinicians are trained. In the modern educational landscape, the mere transmission of factual information is no longer sufficient. Medical education must equip learners with the ability to integrate, apply, and retain knowledge in clinically meaningful ways. This is particularly critical in the surgical specialities, where understanding intricate anatomical relationships forms the cornerstone of procedural competence and patient safety. Among these, otorhinolaryngology (ENT) poses a distinct challenge due to the compact yet complex anatomical zones it encompasses, necessitating both high-level spatial reasoning and the ability to apply anatomical knowledge in dynamic clinical contexts [1,2].

Despite curricular reforms and the shift toward Competency-Based Medical Education (CBME), anatomy teaching in many institutions remains heavily reliant on passive delivery methods: PowerPoint-based lectures, textbook references, and limited cadaveric exposure. Such methods, while foundational, may often disengage learners and fail to scaffold higher-order cognitive skills sufficiently. The disconnect between theoretical instruction and its real-world application has been widely recognised as a barrier to effective learning, particularly in domains like surgical anatomy, where visual-spatial cognition, tactile understanding, and interpretative reasoning are deeply intertwined [1,3,4].

Globally, educational research has responded to this challenge through the development of blended, simulation-enhanced, and student-centred pedagogies that engage multiple domains of learning. These include digital tools such as 3D visualisation platforms, mannequin-based procedural simulation, and task-based collaborative activities that simulate clinical reasoning in a low-risk environment. Each of these modalities has independently demonstrated improvements in knowledge acquisition, learner engagement, and perceived confidence [5,6]. Yet, integrated approaches that combine these elements into a structured, outcome-driven teaching-learning strategy remain underutilised in the undergraduate setting, especially in resource-constrained or traditional curricula.

In this context, the Fusion Teaching-Learning Method (TLM), conceptualised as “From Pixels to Scalpels,” represents a pedagogical innovation designed to operationalise active learning across the cognitive spectrum. This method integrates three key elements: (i) Pixel-based digital visualization, leveraging 3D anatomy apps, animations, and videos to establish conceptual foundations; (ii) Scalpel-based hands-on engagement through anatomical models, mannequins, and procedural kits to reinforce practical understanding; and (iii) Collaborative team-based tasks, including case-based scenarios and clinical rubrics, to cultivate critical thinking, communication, and decision-making aligned with Bloom’s higher-order taxonomy. By offering repeated, multi-sensory engagement with the content, the method targets durable learning and promotes transferability to clinical environments [5,7].

The metaphor “From Pixels to Scalpels” is not merely rhetorical; it captures a deliberate shift from abstract, digital interaction to tangible, applied competence. This progression reflects the principles of cognitive constructivism, which posits that learning occurs most effectively when learners actively construct meaning through interaction, reflection, and feedback. In line with CBME, the fusion model also supports holistic competency development, including analytical reasoning, teamwork, and communication competencies often under-addressed in didactic settings [8].

While individual components of blended or simulation-based learning have been validated in various studies, there is a notable paucity of evidence regarding their collective integration as a unified teaching-learning strategy within ENT education at the undergraduate level. Moreover, limited Indian data is evaluating how such fusion methodologies impact not only immediate comprehension but also long-term retention and real-world application [9].

The present study, conducted in a structured crossover observational design, was therefore conceived with two central aims. First, to evaluate whether the Fusion TLM improves comprehension, knowledge retention, and clinical application of ENT surgical anatomy in MBBS Phase III Part I students when compared to traditional teaching methods. Second, to assess student perceptions regarding its usefulness, relevance, and engagement value, thereby capturing both outcome and experiential dimensions of the intervention. By generating empirical evidence through quantitative testing and qualitative feedback, this study seeks to contribute to a growing body of work advocating for transformative pedagogies in undergraduate medical education.

## Materials and methods

This prospective, crossover observational study was conducted over a three-month period (October to December 2024) in the Department of Otorhinolaryngology at Malla Reddy Medical College for Women (MRMCW), Hyderabad, India. The primary objective was to evaluate the comparative effectiveness of an innovative Fusion-TLM, titled “From Pixels to Scalpels,” against the conventional lecture-based approach in improving comprehension, knowledge retention, and clinical application of complex ENT surgical anatomy. The study was also designed to capture the perceptions of undergraduate learners regarding the pedagogical utility and relevance of this multimodal teaching strategy.

A total of 60 medical students from MBBS Phase III Part I (2021 batch), all of whom were attending their scheduled ENT clinical postings, were recruited for the study through a simple random sampling method. Each student provided written informed consent prior to participation, and the study protocol received ethical clearance from the Institutional Ethics Committee (Approval Number: MRMCWIEC/AP/308/2024, dated 08/10/2024). The inclusion criteria comprised students who were willing to participate in both arms of the teaching-learning intervention. Those who declined to provide consent or were absent during critical stages of assessment were excluded from the study.

Participants were randomly divided into two equal groups: Group A and Group B, each comprising 30 students. A crossover instructional design was implemented across two ENT anatomy topics. Group A received Topic I through the Fusion TLM and Topic II via the Traditional TLM, while Group B underwent the reverse sequence. This design allowed each student to experience both teaching methodologies, enabling intra-individual comparison while minimising potential instructional bias.

The Fusion TLM was implemented in a structured and staged format. It commenced with a 30-minute session of digital learning ("Pixels"), utilising high-resolution 3D anatomical visualisation tools, animated video content, and interactive simulations aimed at fostering conceptual clarity. This was followed by a 15-minute hands-on module ("Scalpels"), which involved anatomical models, mannequins, and tactile demonstrations to reinforce psychomotor skills. After these foundational sessions, students were grouped into teams of six and assigned task-based collaborative learning activities designed around clinical scenarios, anatomical charts, and digital presentations. These team tasks were submitted and presented after a one-week interval and were evaluated using a structured rubric aligned with Bloom’s taxonomy, encompassing traits such as applied reasoning, presentation quality, clinical depth, and innovation. Each session concluded with a 10-minute interactive discussion, during which facilitators clarified doubts, provided feedback, and encouraged reflective learning. The rubric was designed by a team of faculty members with experience in medical education and aligned to Bloom’s taxonomy to ensure content and construct validity. While it was not formally validated statistically, it underwent peer review within the department for face clarity and relevance, ensuring practical feasibility and consistency across assessments.

In contrast, the Traditional TLM relied on conventional approaches. A 30-minute didactic PowerPoint presentation (Microsoft® Corp., Redmond, WA, USA) was delivered by the faculty, followed by 20 minutes of self-study using printed handouts, 15 minutes of group discussion among peers, and a final 10-minute session dedicated to addressing doubts. This method aimed to replicate standard classroom teaching practices commonly used in Indian medical colleges, serving as a comparative control for the new instructional model.

The study employed a combination of quantitative and qualitative assessment strategies. For quantitative analysis, all participants underwent structured multiple-choice question (MCQ)-based tests, designed with both factual and clinical scenario items. These assessments were conducted in three phases: a pre-test (administered before the topic was delivered), a post-test (administered immediately after the topic was completed), and a retention test (administered two weeks later: this two-week interval was chosen based on prior literature suggesting it is sufficient to evaluate intermediate-term memory retention without undue influence from short-term recall or long-term forgetting curves. It also allowed alignment with the academic schedule and minimised attrition bias from prolonged follow-up.). These tests were used to measure knowledge acquisition, immediate comprehension, and long-term retention, respectively. In addition to individual assessments, team-based performance was evaluated after the group task presentations using a rubric constructed to reflect various cognitive levels defined in Bloom’s taxonomy, including comprehension, application, analysis, and creativity.

For qualitative evaluation, student perceptions were collected using a structured Likert-scale questionnaire [10] administered at the end of both instructional phases. The survey captured perceptions on various domains, such as instructional clarity, engagement, relevance, ability to integrate knowledge clinically, and preference for future learning. The aim was to triangulate objective performance data with subjective learner experience to provide a comprehensive understanding of instructional impact.

All data were analysed using IBM SPSS Statistics for Windows, Version 23 (Released 2016; IBM Corp., Armonk, New York, United States). Continuous variables, including test scores and rubric grades, were presented as means and standard deviations. Paired sample t-tests were applied to compare within-group changes from pre- to post-test and retention phases. Between-group comparisons for team assessment outcomes were conducted using unpaired t-tests. Perception data were analysed descriptively using frequencies and percentages. A two-tailed p-value of less than 0.05 was considered statistically significant for all inferential tests.

## Results

All 60 MBBS Phase III Part I students completed the study in its entirety, and data from both instructional arms were analysed to evaluate the comparative effectiveness of the Fusion-TLM versus traditional instructional modalities. The analysis was conducted across four core domains: immediate knowledge acquisition, applied understanding via rubric-based team assessments, long-term retention of knowledge, and learner perceptions of the pedagogical model.

Knowledge acquisition through pre- and post-test assessments

Baseline knowledge levels, assessed via pre-tests, did not show any statistically significant difference between the two groups. In Pre-test 1, the mean scores of Group A and Group B were 4.07 and 4.70, respectively, with a p-value of 0.12, indicating comparable foundational understanding before intervention. Similarly, Pre-test 2 scores (7.23 vs. 7.37; p = 0.838) confirmed equivalent starting knowledge in the second instructional cycle.

However, significant differences emerged in post-test performance. In Post-test 1, students who underwent the Fusion TLM achieved a markedly higher mean score of 14.00 ± 2.50, in contrast to 12.00 ± 3.00 among those taught by traditional methods (t = 2.805, p = 0.007). A similar pattern was observed in Post-test 2, with Fusion-trained students scoring 14.00 ± 2.00, outperforming the traditional group, which scored 12.25 ± 2.50 (t = 3.000, p = 0.004). These results clearly indicate that the Fusion approach significantly enhanced immediate comprehension and conceptual grasp of complex ENT surgical anatomy (Table 1).

**Table 1:** Pre- and Post-test Score Comparison Between Fusion and Traditional Methods SD: standard deviation

Test Phase	Mean (A Batch)	Mean (B Batch)	SD (A Batch)	SD (B Batch)	t-test	p-value
Pre-test 1	4.0667	4.7000	1.43679	1.66402	1.578	0.12
Post-test 1	14.0002 (Fusion Method)	12.0011 (Traditional)	2.5003	3.0012	2.805	0.007
Pre-test 2	7.2333	7.3667	2.69972	2.31164	0.206	0.838
Post-test 2	12.2481 (Traditional)	14.0003 (Fusion Method)	2.5001	2.0023	3.000	0.004

Application-based performance via rubric assessment

To evaluate applied learning and collaborative performance, students were assessed through structured rubric grading. For Topic I, the group taught using the Fusion TLM achieved a substantially higher mean rubric score of 9.85 ± 6.75, compared to 6.05 ± 3.58 in the traditional arm (t = 3.145, p = 0.003). This performance trend was mirrored in Topic II, where the Fusion cohort scored 8.93 ± 6.17, significantly exceeding the 5.45 ± 2.78 obtained by the traditional method group (t = 3.249, p = 0.002). These results reinforce the utility of the Fusion method in promoting higher-order thinking, critical reasoning, and collaborative engagement among medical students (Table 2).

**Table 2:** Rubric Grading Scores for Team Assessment TLM: Teaching-Learning Method

Topic	Groups	N	Mean	Std. Deviation	t-test	p-value
I	A Batch (Fusion-Based TLM)	30	9.85	6.75	3.145	0.003
	B Batch (Traditional TLM)	30	6.05	3.58		
II	A Batch (Traditional TLM)	30	5.45	2.78	3.249	0.002
	B Batch (Fusion-Based TLM)	30	8.93	6.17		

Knowledge retention two weeks post-intervention

The retention tests conducted two weeks after each instructional cycle revealed a sustained cognitive advantage among students exposed to the Fusion TLM. In Retention Test 1, the Fusion group achieved a mean score of 15.15 ± 2.50, compared to 13.00 ± 2.80 in the traditional group (t = 3.130, p = 0.003). Similarly, in Retention Test 2, students who had undergone the Fusion learning intervention scored 14.50 ± 2.50, significantly higher than the 12.01 ± 3.02 recorded in the traditional group (t = 3.476, p = 0.001). These findings confirm that the Fusion TLM not only enhances immediate understanding but also promotes more durable retention of anatomical knowledge over time (Table 3).

**Table 3:** Knowledge Retention Test Scores (Two Weeks Post-TLMs) TLM: Teaching-Learning Method

Topics	Mean (A Batch)	Mean (B Batch)	Std. Deviation (A)	Std. Deviation (B)	t-test	p-value
Retention Test - 1	15.1527 (Fusion Method)	13.0067 (Traditional Method)	2.5006	2.8011	3.130	0.003
Retention Test - 2	12.0107 (Traditional Method)	14.5000 (Fusion Method)	3.0200	2.5024	3.476	0.001

Student perception and acceptability of fusion TLM

Feedback from students gathered through a 5-point Likert scale showed overwhelming support for the Fusion model. All students (100%) either agreed or strongly agreed that the Fusion TLM enhanced their motivation and engagement, with 52% strongly agreeing. Regarding knowledge retention, 95% responded positively, while 86% affirmed improved clinical application of surgical anatomy, including 48% who selected “strongly agree.”

Furthermore, 90% of students agreed that the Fusion TLM effectively bridged the gap between theoretical knowledge and practical application, and a similar majority acknowledged the role of pixel-based tools in enhancing their spatial understanding before transitioning to hands-on models. In terms of overall impact, 53% strongly agreed that the Fusion method was more effective than traditional instruction. Most notably, 75% strongly agreed that the technique improved their self-confidence, a key determinant in the transition from knowledge to competence (Table 4).

**Table 4:** Student’s Perception of Fusion TLM - Likert Scale Distribution (% Responses) TLM: Teaching-Learning Method

Statement	Strongly Disagree	Disagree	Neutral	Agree	Strongly Agree
Fusion TLM increases self-motivation and engagement	0 (0%)	0 (0%)	0 (0%)	29 (48%)	31 (52%)
Fusion TLM helps in retaining knowledge	0 (0%)	3 (5%)	0 (0%)	34 (57%)	23 (38%)
Fusion TLM helps in the clinical application of surgical anatomy	0 (0%)	0 (0%)	10 (17%)	21 (35%)	29 (48%)
Fusion TLM bridges theory and practice	0 (0%)	6 (10%)	0 (0%)	29 (48%)	25 (42%)
Pixel-based approach enhances understanding	0 (0%)	4 (7%)	0 (0%)	30 (50%)	26 (43%)
Fusion TLM increased understanding of ENT surgical procedures	0 (0%)	3 (5%)	0 (0%)	27 (45%)	30 (50%)
Fusion TLM enhances understanding of complex ENT surgical anatomy	0 (0%)	1 (2%)	0 (0%)	29 (48%)	30 (50%)
Fusion TLM is more effective than traditional TLM	0 (0%)	0 (0%)	0 (0%)	28 (47%)	32 (53%)
Fusion TLM engages and improves understanding of surgical anatomy	0 (0%)	3 (5%)	0 (0%)	22 (37%)	35 (58%)
Fusion TLM helps in building self-confidence	0 (0%)	0 (0%)	0 (0%)	15 (25%)	45 (75%)

## Discussion

The present study provides persuasive evidence that the Fusion TLM, an integrated pedagogical approach combining pixel-based visualisation, scalpel-based hands-on reinforcement, and collaborative team-based application, offers significant advantages over traditional methods in enhancing the comprehension, retention, and clinical translation of complex ENT surgical anatomy. By triangulating quantitative data from objective tests and rubric-based assessments with qualitative learner feedback, this study affirms that multimodal, constructivist teaching strategies are not only feasible in undergraduate medical settings but also pedagogically superior in both cognitive and affective outcomes.

The marked improvement in post-test scores following the Fusion TLM intervention highlights its impact on short-term knowledge acquisition, particularly in domains that demand high-level spatial reasoning and anatomical precision. This finding is consistent with earlier work, which noted that anatomical visualisation tools, such as 3D models and virtual dissection platforms, enhance learner engagement and mental mapping, especially in surgical disciplines [7]. Similarly, Shapiro et al. (2020) demonstrated that combining visual and tactile sensory pathways facilitates deeper encoding and recall of anatomical structures, supporting the dual-coding theory of learning [11]. In the current study, students exposed to Fusion TLM consistently outperformed their peers in both ENT topics, despite comparable baseline knowledge, suggesting that the method does more than merely review content; it reconstructs the learning experience in a cognitively rich and clinically relevant format.

Beyond knowledge acquisition, the significantly higher rubric scores in the Fusion group assessing presentation skills, depth of understanding, application of clinical knowledge, and innovation highlight the strength of this method in fostering applied learning. The design of team-based, scenario-driven assignments in the Fusion arm appears to have promoted engagement with complex surgical reasoning, echoing findings by Dnyanesh et al. (2024), who emphasised that collaborative, task-based learning promotes critical thinking and retention of clinically applicable knowledge. The assessment framework employed in this study, aligned with Bloom’s taxonomy, further ensured that students were evaluated not merely on recall but on their ability to analyse, apply, and communicate their anatomical understanding, a crucial expectation in CBME [12]. Importantly, the improvement in rubric-based scores among Fusion TLM participants, particularly in domains such as applied reasoning, clinical depth, and innovation, reflects a measurable enhancement in clinical translation of anatomical knowledge. These traits map directly onto real-world competencies, including surgical orientation, instrument handling awareness, and decision-making in ENT scenarios. Although the study did not include direct psychomotor skill assessment (e.g., via an Objective Structured Clinical Examination (OSCE)), the team-based tasks and rubric structure were designed to simulate objective skill components such as spatial orientation, clinical communication, and procedural planning. Thus, the model supports not only cognitive outcomes but also the foundational layers of procedural competence, bridging the gap between theoretical understanding and actionable clinical skills.

Perhaps most significantly, the study demonstrated that knowledge retention two weeks after the intervention was markedly higher in the Fusion TLM group. This aligns with findings from Antipova et al. (2024), who reported that multimodal instruction improves long-term memory consolidation by stimulating deeper cognitive processing and by enabling more robust neural encoding pathways [13]. The delayed testing used in this study served as an objective indicator of the durability of learning, an area often neglected in single-session instructional research. The sustained advantage observed in both retention tests suggests that the repeated, multisensory exposure offered by the Fusion model creates a richer and more resilient cognitive scaffold compared to isolated lecture-based instruction [14].

Another critical dimension of the study lies in student perception, which offers insights into the affective and motivational impact of the instructional method. The overwhelmingly positive responses, 100% agreement that the method increased motivation and engagement, and 75% affirming improvement in self-confidence, mirror findings from contemporary literature emphasising learner autonomy and satisfaction in active learning environments. For instance, a study by Jahromi et al. (2024) concluded that learners perceive 3D visual tools and integrated simulation strategies as not only more engaging but also more effective in linking basic science to clinical practice. The constructivist principle underlying the Fusion TLM, which places learners at the centre of the instructional process, likely contributed to this favourable reception. By allowing students to interact with the content through multiple sensory modalities and by encouraging self-directed exploration and team collaboration, the Fusion method aligns closely with adult learning theory and the principles of self-determined learning (heutagogy) [15].

The high proportion of students who agreed that the Fusion method helped bridge the gap between theory and practice (90%) is particularly relevant in today’s curricular discourse. Despite the implementation of CBME in Indian medical colleges, traditional anatomy instruction continues to face criticism for its fragmentation and lack of clinical relevance. A similar study has emphasised the need to embed clinical context into basic science education to improve relevance, motivation, and transferability. The present study operationalised this integration not only through case-based learning and rubric-aligned assessments but also by sequencing the learning from digital “pixels” to hands-on “scalpels,” thus literally and metaphorically guiding learners from abstraction to application [16].

It is also noteworthy that the Fusion TLM demonstrated its efficacy within a resource-constrained institutional setting, a factor that enhances the model’s scalability. While digital tools and mannequins may require initial investment, many of the interventions used (e.g., animations, collaborative discussions, chart-based tasks) are low-cost and adaptable to varied institutional contexts. This aligns with findings from Vallée et al. (2020), who demonstrated that blended learning approaches can be successfully adapted to low-resource environments without compromising educational outcomes [17].

Nonetheless, the study's strengths are notable. The design was procedurally stringent, incorporating both subjective and objective data points, triangulated across immediate, applied, and delayed learning outcomes. The alignment with CBME principles and Bloom’s taxonomy enhances its pedagogical validity, and the integration of student feedback ensures that the approach is learner-informed and contextually grounded. Looking forward, the implications of this study are significant. In an era where medical education is transitioning from volume-based teaching to competency-focused frameworks, the Fusion TLM offers a feasible, evidence-based model for teaching anatomy in a clinically integrated and student-centred manner. Moreover, its design inherently promotes vertical integration, teamwork, and reflective practice, core competencies expected of 21st-century physicians.

However, some limitations merit consideration. First, the study was limited to a single institution and speciality, which may restrict generalizability. Future research involving multi-centre trials across different anatomical domains would be valuable to establish broader applicability. Second, while the crossover design controlled for intergroup variability, there may have been residual confounding due to topic complexity or facilitator influence, although efforts were made to standardise content delivery. Third, while retention was measured at two weeks, it remains unknown whether these gains persist over months or translate into clinical performance during internships or postgraduate training. Fourth, the study did not include long-term follow-up assessments (e.g., during internships or clinical rotations), which would have allowed evaluation of sustained knowledge retention and real-world clinical transfer. Fifth, no direct correlation was assessed between Fusion TLM exposure and objective clinical performance in ENT procedures, such as through OSCEs or surgical skills labs. Future studies may consider longitudinal tracking or integrating structured skill assessments to evaluate the translational impact of such pedagogical models more comprehensively.

## Conclusions

This study substantiates the pedagogical value of the Fusion TLM, demonstrating its superiority over traditional instructional approaches in enhancing immediate comprehension, long-term retention, and clinical application of ENT surgical anatomy among undergraduate medical students. By integrating pixel-based digital tools, hands-on anatomical engagement, and collaborative clinical reasoning tasks, the method fostered a multi-sensory, constructivist learning environment aligned with the goals of CBME. Quantitative data revealed statistically significant improvements in knowledge acquisition and retention, while rubric-based assessments affirmed the development of higher-order cognitive skills. The crossover design further strengthened internal validity by enabling within-subject comparisons across both topics and methods.

Equally significant were the overwhelmingly positive student perceptions, which underscored heightened engagement, enhanced spatial understanding, and increased self-confidence - key determinants of educational success and clinical readiness. The method’s scalability within resource-limited institutional settings, coupled with its alignment to Bloom’s taxonomy and adult learning principles, marks it as both feasible and pedagogically robust. While the study’s scope was limited to a single institution and speciality, the outcomes offer strong justification for broader implementation and longitudinal research across varied disciplines. In sum, the “From Pixels to Scalpels” model presents a compelling blueprint for anatomically immersive, clinically relevant, and learner-centred medical education.
